# A girl with a torticollis without trauma: Grisel's syndrome

**DOI:** 10.11604/pamj.2014.19.371.5846

**Published:** 2014-12-11

**Authors:** Ozgur Sogut, Kenan Ahmet Turkdogan

**Affiliations:** 1 Bezmialem Vakif University, Faculty of Medicine, Department of Emergency Medicine, Istanbul, Turkey

**Keywords:** Grisel′s syndrome, atlanto-axial subluxation, painful neck

## Image in medicine

A 7 year-old girl patient was referred from pediatric emergency department (ED) to our emergency department due to cervical pain and neck stiffness. On her physical examination, torticollis was found in the neck. There was no history of trauma. Physical examination showed no focal neurological deficits. On further anamnestic evaluation the patient's parents revealed that she had presented to paediatric ED due to throat and neck pain and put on antibiotics therapy for 5 days. A computed tomography (CT) scan of the patient's neck was performed. A cervical lymphadenopathy associated with atlanto-axial subluxation shadow. The atlas was rotated on one articular process with 3-5 mm anterior displacement, compatible with Type II subluxation. Non-traumatic or inflammatory atlanto-axial subluxation is known as Grisel's syndrome. The subluxation was stabilized with external stabilization (rigid cervical collar). Skeletal muscle relaxants, antibiotics and nonsteroidal anti-inflammatory medications with bed rest were given for further treatment. Early diagnosis of Grisel's syndrome is of crucial importance due to the neurological deficits suc as recurrence or permanent neck deformity. CT, magnetic resonance imaging (MRI) or other imaging tests are required to demonstrate fractures and displaced bone fragments (ie., atlanto-axial subluxation). The primary treatment of early detected Grisel's syndrome is conservative including antibiotic therapy, bed rest, muscle relaxants, external fixation and anti-inflammatory therapy.

**Figure 1:**
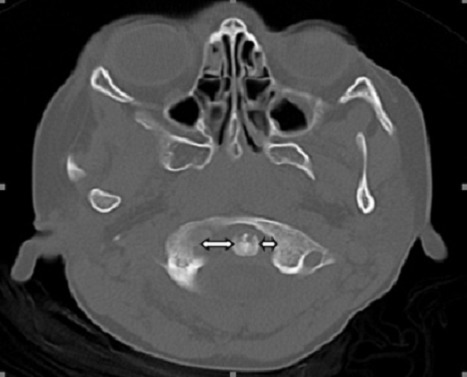
The atlas was rotated on one articular process with 3-5 mm anterior displacement, compatible with type II subluxation in patient's computed tomography

